# Mental health help-seeking intentions among health workers in the east coast of peninsular Malaysia: Perceived barriers and predictive factors

**DOI:** 10.1371/journal.pone.0344007

**Published:** 2026-03-03

**Authors:** Muhammad Syafiq Kunyahamu, Aziah Daud, Ijlal Syamim Mohd Basri, Tengku Alina Tengku Ismail, Mohd Faiz Md Tahir

**Affiliations:** 1 Jerantut District Health Office, Pahang, Malaysia; 2 Department of Community Medicine, School of Medical Sciences, Universiti Sains Malaysia Health Campus, Kelantan, Malaysia; 3 Department of Psychiatry and Mental Health, International Islamic University, Kuantan, Malaysia; Universiti Putra Malaysia, MALAYSIA

## Abstract

**Introduction:**

Mental health problems among health workers are a growing concern globally, including in Malaysia. Despite the availability of mental health services, some health workers do not seek professional help. This study aims to determine the level of health workers’ intention to seek professional help, examine the barriers they perceive, and identify predictors of mental health help-seeking intention.

**Methods:**

This cross-sectional study involved 470 health workers in the East Coast region of Peninsular Malaysia. Data was collected using a self-administered questionnaire. Linear regression analysis was employed to identify the predictors of professional help-seeking intention.

**Results:**

The mean score for mental health help-seeking intention was 4.90 (SD = 1.03). Perceived need for help positively predicted help-seeking intention (B = 0.532, p < 0.001), while perceived stigma barriers negatively predicted help-seeking intention (B = −0.588, p < 0.001). Notable barriers perceived included concerns about perceptions of weakness, feelings of embarrassment, a preference for handling problems independently, and challenges in taking time off work.

**Conclusions:**

This study highlights the roles of the perceived need for help and perceived stigma barriers in predicting health workers’ help-seeking intentions, offering a basis for targeted interventions and policies to enhance mental health support within Malaysian healthcare settings.

## Introduction

Mental health-related problems can occur to anyone, regardless of age, ethnicity, sex, occupation, or socioeconomic background. Health workers, which includes clinical and nonclinical staff, can also face problems with their mental health. Psychological stress, anxiety, and depression are common examples of health workers’ mental health-related problems. Some have even considered suicide [1–4]. Several studies have provided insight into why health workers are more prone to mental health problems. This could possibly be due to their exposure to several factors that have a detrimental effect on their mental health, such as heavy workloads, poor social support, long working hours, conflict between colleagues, and workplace violence [5–7].

Seeking help from professional mental health services is key to addressing and managing these problems. Seeking appropriate help is viewed as a protective factor that may reduce the detrimental consequences of mental health problems [8]. However, despite their exposure to health-related information and the presence of mental health support services in healthcare facilities, studies have revealed that some health workers who are experiencing mental health problems do not seek professional help [9–12]. They encounter various types of barriers in regard to accessing mental health support services, such as experiencing stigma, having a lack of awareness about available services, facing time constraints, and fearing potential job-related consequences, which can lead to a reluctance or lack of intention to seek help when it is needed [11,13–15].

Mental health help-seeking intentions refer to a person’s conscious plan to seek help when they are facing a mental health problem. This intention is driven by the desire to reduce their distress and obtain psychological support [16]. Help-seeking intention plays a crucial role in the help-seeking process and can significantly influence whether individuals will seek help when they need it. A lack of interest in seeking help can prevent people from using available mental health services. People are unlikely to utilise mental health services if they are not interested in seeking help for mental health problems [17]. Understanding the factors that influence health workers’ help-seeking intentions is important for designing effective interventions, improving mental health outcomes, enhancing workers’ well-being and productivity, reducing stigma, and contributing to the overall effectiveness of the healthcare system.

Even though this is an important issue, our understanding of health workers’ intentions to seek professional help remains limited, particularly in the Malaysian context. Previous research has identified several predictors of help-seeking intention, such as sociodemographic characteristics, individual attitudes, mental health literacy, and stigma [18–22]. However, most of these studies have been conducted in Western countries, and their findings may not be directly applicable to the Malaysian health worker context due to cultural, social, and healthcare system differences. This gap in the literature is significant, considering the important role that health workers play in healthcare delivery and the potential impact of their mental well-being on patient care.

While stigma and perceived need are frequently reported predictors of professional help-seeking in international literature, the Malaysian context may shape these relationships through local sociocultural and workplace factors. In Malaysia, mental illness has been described as highly stigmatized, including perceptions of “weakness” and discrimination that can affect social and employment outcomes. Such stigma may be especially salient for healthcare workers, who may worry about confidentiality, professional reputation, and career implications when seeking care. Therefore, examining predictors and barriers within Malaysian healthcare settings provides locally relevant evidence to inform workplace mental health strategies [23,24].

Considering the limited evidence in Malaysia, this study aims to determine the level of health workers’ intention to seek professional mental health help, examine perceived barriers, and identify predictors of help-seeking intention among health workers in East Coast Region of Peninsular Malaysia.

## Method

### Participants and procedures

This cross-sectional study was conducted from 1^st^ of March till 15^th^ of July 2023, involving 470 health workers who were randomly selected in Pahang, one of the East Coast states of Peninsular Malaysia. The required sample size was calculated using a single-mean formula, with a standard deviation (σ) of 1.48, as derived from previous research [25]. The precision of estimation (Δ) was set at 0.15, and the Z value corresponding to a 95% confidence interval (Z(1- α/2)) was set at 1.96. Based on these parameters, the calculated sample size was 374. After anticipating a 20% nonresponse rate, this study required at least 468 respondents.

A multistage sampling method was employed to ensure that the research sample was representative of the reference population. Initially, three districts were selected namely Kuantan, Jerantut and Temerloh using a simple random sampling method. Subsequently, healthcare facilities within these districts were categorized into three distinct workplace settings: hospital, health clinic, and general practitioner (GP). A stratified proportionate sampling was then employed to determine the required number of participants from each workplace setting. From each category, facilities were randomly selected. Within each chosen facility, respondents were then randomly selected from the health workers list. Respondents were selected based on the following inclusion criteria: workers who were over 18 years old, had been working for at least a year and were good at reading and understanding the Malay language. Those with conditions that hindered their ability to understand or complete the questionnaires were excluded from the study.

### Measures

Participants were recruited through email invitations, which included a brief explanation of the study’s purpose, confidentiality assurance, and the voluntary nature of participation. They were provided with a link to an online structured, self-administered questionnaire and informed that their responses would remain anonymous to minimize bias. An electronic participant information sheet was displayed on the first page of the online questionnaire, and informed consent was obtained electronically before respondents could proceed to the survey. The questionnaire was divided into several sections: Section A collected sociodemographic information such as age, sex, ethnicity, marital status, education level, and monthly income. Section B assessed the perceived need for help and previous help-seeking experiences. Section C explored mental health help-seeking intentions, and Section D assessed perceived barriers to seeking professional mental health help. To ensure data quality, the online form restricted submissions to one response per email address and required completion of all mandatory items to minimize missing data. Structured response formats and built-in validation checks were used to enhance completeness and consistency of responses.

### Perceived need for help and previous help-seeking experience

Two single-item questions were adapted from a local study by [26] to assess whether the respondents had ever thought they might need professional help for emotional or mental health concerns (perceived need for help) and whether they had previously received or sought any professional help. All of these items were responded to as 0 (No) or 1 (Yes).

### Professional mental health seeking intention

The intention to seek help from mental health professionals was assessed using the Mental Health Seeking Intention Scale (MHSIS), which was used in this study with permission from the original author [27]. This three-item questionnaire uses a 7-point Likert scale, ranging from 1 (Strongly Disagree) to 7 (Strongly Agree), to assess an individual’s intention to consult mental health professionals if they experience a mental health concern. The MHSIS has demonstrated good reliability and validity. The scale demonstrated good internal consistency in this study, with a Cronbach’s alpha of 0.791.

### Perceived barriers to professional help-seeking

Perceived barriers to mental health help-seeking were assessed using the validated Malay version of the Barriers to Access to Care Evaluation scale (MBACE). The scale consists of 28 items, with 12 items specifically designed to assess stigma-related barriers and the remaining 16 items focused on non-stigma-related barriers. Participants responded to each item on a 4-point Likert scale from 0 (not at all) to 3 (a lot), indicating how much each item listed might prevent or discourage them from seeking professional help during emotional challenges. The MBACE was previously translated and validated with permission from the original author, as reported in a prior publication and has good internal consistency and construct validity [28]. In this study, the scale exhibited a Cronbach’s alpha value of 0.89, indicating good reliability in our sample.

### Statistical analysis

Data were analyzed using IBM SPSS software, version 26 [29]. The initial exploration assessed for missing values and the distribution of numerical variables. Continuous variables were represented by the mean and standard deviation (SD), and categorical variables were represented by the frequency and percentage. Data cleaning resulted in 470 respondents being included in the analyses. The MHSIS mean intention score was calculated by summing the individual scores for all three items in the MHSIS and dividing by three. Higher scores indicate greater intention. For the MBACE, mean scores were computed separately for the stigma and non-stigma subscales by summing the scores of individual items within each subscale and then dividing by the number of items in that subscale. Higher scores indicate more perceived barriers to seeking mental health help.

A linear regression model was used to identify the predictors of professional help-seeking intention. The dependent variable was the mean MHSIS score. The independent variables were sex, age, ethnicity, educational level, income, marital status, previous mental health help-seeking experiences, perceived need for help, perceived stigma-related barriers, and perceived non-stigma-related barriers. Independent variables with a p-value < 0.25 in simple linear regression were included in the subsequent multiple linear regression analysis. Variable selection in the multiple linear regression models was conducted using a stepwise process, including backward and forward selection procedures. At this stage, a preliminary main effect model was obtained. All possible interactions between significant variables in the preliminary main effect model were checked. Multicollinearity was assessed using variance inflation factors (VIF), with a threshold of 10, to ensure that the predictors in the model were not highly correlated. The fitness of the final model was also assessed to ensure that it provided a good fit to the data, as indicated by the R^2^ value.

### Ethical clearance

This study received ethical approval from the Malaysia Research Ethics Committee (MREC) of the Ministry of Health Malaysia (NMRR ID-22–01529-H11, Date: 22 September 2022) and the Human Research Ethics Committee USM, Malaysia (USM/JEPeM/22070479, Date: 12 October 2022). All procedures followed the most recent version of the 1975 Declaration of Helsinki for medical research. Informed consent was provided by all respondents before they participated in the study. Minors were not included in our study. Confidentiality of data was guaranteed, with access to the data strictly limited to the researchers.

## Results

### Characteristics of the respondents

Out of the 470 respondents, 71.9% were female, and 28.1% were male. The respondents had a mean age of 37.2 years. The sample was predominantly Malay (94.3%). Regarding the perceived need for professional help for mental health concerns, almost half of the respondents (49.4%) reported that they had felt such a need, regardless of their current mental health status. This indicates that a considerable proportion of health workers in our sample recognized a potential need for mental health support, whether in the past, present, or anticipated future. The general characteristics of the respondents are summarised in Table 1.

**Table 1:** Descriptive characteristics of the respondents (n = 470).

Variables	n (%)	mean (SD)
Age (Years)		37.2 (6.92)
Sex		
Female	338 (71.9)	
Male	132 (28.1)	
Ethnicity		
Malay	443 (94.3)	
Non-Malay	27 (5.7)	
Education Level		
Secondary Education	135 (28.7)	
Diploma	196 (41.7)	
Bachelor degree	122 (26)	
Master/PhD	17 (3.6)	
Monthly Income		
Less than RM 4850	319 (67.9)	
RM 4850–10,959	131 (27.9)	
> RM10,960	20 (4.3)	
Marital Status		
Unmarried ^a^	71 (15.1)	
Married	399 (84.9)	
Worker Category		
Clinical worker	358 (76.2)	
Nonclinical worker	112 (23.8)	
History of Mental Health Diagnosis		
Yes	14 (3.0)	
No	456 (97.0)	
Past Mental Health Help-Seeking Experience		
Yes	51 (10.9)	
No	419 (89.1)	
Perceived Need for Help		
Yes	232 (49.4)	
No	238 (50.6)	

RM – Ringgit Malaysia; ^a^ – Single or Separated.

### Mental health help-seeking intentions

The MHSIS scores ranged from 2.67 to 7.00, with a mean of 4.90 (SD = 1.03). This mean score indicates a moderate to high level of intention among health workers to seek professional mental health help. This suggests that, on average, the respondents somewhat intended to seek help if they were experiencing mental health problems.

### Perceived barriers to professional mental health help-seeking

In our study, the mean score for stigma-related barriers, as measured by the MBACE, was 1.17 (SD = 0.60). For non-stigma-related barriers, the mean score was 0.93 (SD = 0.40). These scores suggest that both types of barriers could potentially discourage health workers from seeking mental health help. Regarding stigma-related barriers, feelings of embarrassment or shame were most commonly perceived as barriers, followed by concerns about other people’s perceptions, fears of being seen as weak, and worries over repercussions on future job prospects. Detailed statistics on the stigma-related barriers can be found in Table 2.

**Table 2:** A description of the perceived stigma-related barriers to help-seeking among participants (n = 470).

Stigma Related Barriers	Not at all n (%)	A little n (%)	Quite a lot n (%)	A lot n (%)
Feeling embarrassed or ashamed.	58(12.3)	175(37.2)	154(32.8)	83(17.7)
Concern about what my friends might think, say or do	85(18.1)	180(38.3)	126(26.8)	79(16.8)
Concern about what people at work might think, say or do	87(18.5)	152(32.3)	152(32.3)	79(16.8)
Concern about what my family might think, say, do or feel	89(18.9)	175(37.2)	152(32.2)	54(11.5)
Concern that I might be seen as weak for having a mental health problem	108(23)	151(32.1)	148(31.5)	63(13.4)
Concern that people might not appreciate me if they found out I was having professional care	121(25.7)	221(47.0)	99(21.1)	29(6.2)
Concern that it might harm my chances when applying for a new job	145(30.9)	162(34.5)	92(19.6)	71(15.1)
Not wanting a mental health problem to be on my medical records	150(31.9)	168(35.7)	73(15.5)	79(16.8)
Concern that peoples I know might find out	152(32.3)	175(37.2)	92(19.6)	51(10.9)
Concern that I might be seen as ‘crazy’	155(33)	178(37.9)	92(19.6)	45(9.6)
Concern that I might be seen as a bad parent	236(50.2)	177(37.7)	46(9.8)	11(2.3)
Concern that my children may be taken into care or that I may lose access or custody without my agreement	258(54.9)	130(27.7)	50(10.6)	32(6.8)

For non-stigma-related barriers, among the commonly perceived as barriers were the desire to solve the problem independently, a belief that the problem would resolve itself, and difficulty taking time off work. Further details can be found in Table 3.

**Table 3:** A description of the Perceived Non-Stigma-Related Barriers to Help-Seeking among participants (n = 470).

Non-Stigma Related Barriers	Not at all n (%)	A little n (%)	Quite a lot n (%)	A lot n (%)
Wanting to solve the problem on my own	24(5.1)	135(28.7)	179(38.1)	132(28.1)
Thinking the problem would get better by itself	66(14.0)	172(36.6)	160(34.0)	72(15.3)
Dislike talking about my feelings, emotions, or thoughts	84(17.9)	154(32.8)	171(36.4)	61(13.0)
Difficulty taking time off work	154(32.8)	152(32.3)	105(22.3)	59(12.6)
Preferring to get help from family or friends	138(29.4)	180(38.3)	104(22.1)	48(10.2)
Concerns about the treatments available (e.g., medication side effects)	194(41.3)	175(37.2)	66(14.0)	35(7.4)
Fear of being put in hospital against my will	240(51.1)	147(31.3)	53(11.3)	30(6.4)
Thinking I did not have a problem	166(35.5)	195(41.5)	85(18.1)	24(5.1)
Preferring to get alternative forms of care (e.g., traditional/religious healing or alternative/complementary therapies)	192(40.9)	192(40.9)	63(13.4)	23(4.9)
Having problems with childcare while I receive professional care	295(62.8)	109(23.2)	47(10.0)	19(4.0)
Problems with transport or traveling to appointments	275(58.5)	133(28.3)	42(8.9)	20(4.3)
Not being able to afford the financial costs involved	288(61.3)	114(24.3)	50(10.6)	18(3.8)
Being unsure where to go to get professional care	207(44)	164(34.9)	85(18.1)	14(3.0)
Having no one who could help me to get professional care	176(37.4)	226(48.1)	54(11.5)	14(3.0)
Thinking that professional care probably would not help	261(55.5)	134(28.5)	64(13.6)	11(2.3)
Previous bad experiences with professional care for mental health	322(68.5)	109(23.2)	33(7.0)	6(1.3)

### Predictors of professional mental health help-seeking intention

The regression analysis showed that only the variables of perceived need for help and perceived stigma barrier were significant predictors of mental health help-seeking intention. The perceived need for help emerged as a significant positive predictor (B = 0.532, t = 6.172, p < 0.001), suggesting that health workers who perceived a greater need for help were more likely to express an intention to seek help from mental health professionals. Conversely, the perceived stigma barrier was a negative predictor of help-seeking intention (B = −0.588, t = −8.167, p < 0.001), indicating that health workers who perceived higher stigma-related barriers were less likely to express an intention to seek help.

The final multiple regression model, which included the perceived need for help and perceived stigma barriers as predictors, accounted for 22% of the variance in help-seeking intention (adjusted R^2^ = 0.22). The R^2^ value also indicates the model’s predictive accuracy. Table 4 shows the outcomes of the regression analysis. The table details the direction of the relationship (either positive or negative) and the level of statistical significance for each variable.

**Table 4:** Simple linear regression and multiple linear regression of professional mental health help-seeking intention among respondents (n = 470).

Variable	SL ^a^		ML ^b^		
	Crude b^c^ (95% CI)	p-value	Adj. b^d^	t-stat	p-value
**Age**	0.009 (−0.005,0.002)	0.199			
**Sex**	−0.061 (−0.267,0.147)	0.567			
**Ethnicity**	−0.249 (−0.650,0.152)	0.223			
**Marital Status**	0.139 (−0.122,0.400)	0.295			
**Educational Level**	−0.011 (−0.124,0.101)	0.846			
**Worker Category**	0.019 (−0.200,0.239)	0.864			
**Income**	0.007 (−0.159, 0.173)	0.933			
**History of Mental Health Diagnosis**	0.199 (−0.351,0.748)	0.478			
**Past Help-Seeking Experience**	0.096 (−0.205, 0.396)	0.531			
**Perceived Need for Help**	0.693 (0.517,0.869)	<0.001	0.532	6.172	<0.001***
**Perceived Stigma Barrier**	−0.690 (−0.833, −0.547)	<0.001	−0.588	−8.167	<0.001***
**Perceived Non-Stigma Barrier**	−0.669 (−0.895, −0.444)	<0.001			

^a^– simple linear regression, ^b^- multiple linear regression, c- crude regression coefficient ^d^- adjusted regression coefficient.

## Discussion

### Mental health help-seeking intentions

The mean score of 4.90 on the MHSIS indicates a moderate to high level of intention to seek help from mental health professionals. This finding demonstrated that, on average, there was a relatively quite good level of help-seeking intention among the health workers participating in this study if they have any mental health concerns. This result is consistent with some other research that also reported a relatively good level of health workers’ intention to seek professional help if they encountered psychological distress [14,30].

Due to their roles and exposure to health-related information, health workers might possess a better level of mental health literacy. This could explain the MHSIS score observed in our sample, as higher mental health literacy often correlates with a stronger intention to seek help [19,22]. Additionally, recent efforts by the Malaysian Ministry of Health, such as the National Centre of Excellence for Mental Health initiation in 2022 and the launch of the “HEAL 15555” hotline, might have boosted mental health awareness, further enhancing the intention to seek help. Furthermore, the COVID-19 pandemic’s influence cannot be overlooked. Its onset not only heightened mental health issues but also magnified societal awareness and made mental health services more accessible [31]. All these factors might explain the observed help-seeking intentions score among the health workers in our study.

However, it is essential to recognise that some of the respondents (17.7%) reported scores below the midpoint of the scale (< 4). This suggests that some health workers have relatively low intentions to seek help, putting them at risk of suffering in silence. While previous studies on mental health professionals indicate a higher likelihood of seeking mental health support, our findings among non-mental health workers suggest similar trends [22]. However, lower help-seeking behavior among certain subgroups indicates a need for tailored interventions. A local study by Chong *et al.*, revealed that two-thirds of civil servants had a low intention to seek mental health counselling, suggesting that low intention to seek help may be a broader issue among Malaysian workers [32].

### Perceived barriers to professional mental health help-seeking

Despite all the efforts to improve mental health awareness and services, health workers still face barriers to mental health help-seeking. This is reflected by the findings of perceived stigma barriers and perceived non stigma barriers among the respondents, which aligns with previous research demonstrating that stigma and non-stigma barriers often deter individuals from seeking help for mental health problems [19,20,22,33].

Common stigma items perceived as barriers in this study, such as concern about being viewed as weak or worried about professional repercussions and feelings of embarrassment, align with findings from other studies among health workers [14,15,34,35]. This suggests that such barriers are common across different populations of health workers. These barriers seem to reflect not only personal fears but also broader societal norms. This is particularly true in Malaysia, where psychological problems are often stigmatised as signs of personal weakness or insanity [24]. Another local study further underscored this by highlighting employment challenges faced by those with a history of mental health problems, noting their struggles to secure employment [23]. Therefore, it is not surprising that some people opt to hide their mental health issues and refrain from seeking professional help to avoid the associated stigma.

Surprisingly, even among health workers who presumably have a good understanding of the importance of professional mental health support, personal attitudes and beliefs emerged as the predominant perceived non stigma barriers to help-seeking in our study. However, these findings are not unique only to our sample population. Previous research found similar patterns among different health worker populations [11,14,33]. This finding suggests the complex relationship between professional knowledge and personal beliefs. It highlights the need for interventions that not only address stigma but also target people’s attitudes and beliefs. Beyond stigma, operational constraints within healthcare settings may also reduce help-seeking, including heavy workload, shift duties, limited flexibility to attend appointments, and practical difficulty obtaining time off. These barriers can persist even when services are available, particularly when accessing care is perceived as disruptive to clinical or administrative responsibilities.

The issue of difficulty taking time off work, as perceived by respondents in our study, aligns with findings from other studies conducted in different populations [10,13–15]. These studies, along with ours, highlight the intense and demanding work schedules faced by health workers, leaving little room for self-care and mental health management. This emphasises the importance of workplace intervention, especially in the healthcare sector, to consider implementing flexible work arrangements and supportive policies to facilitate access to mental health services among their employees.

### Predictors of help-seeking intention

Our study provides additional support for the evidence that perceived stigma barriers and perceived need for help significantly influence health workers’ intentions to seek mental health professional help. Specifically, the role of perceived need for help as a positive predictor aligns with findings from other studies [36,37]. These results emphasise the need to enhance awareness among health workers about the recognition of their own mental health needs, and potential stigma, as this could be a key strategy to encourage mental health help-seeking.

Despite the significant role of the perceived need for help in predicting help-seeking intentions, 50% of respondents did not perceive a need for mental health help. However, the lack of perceived need among our respondents may reflect that they truly do not ever have psychological health issues at all, or it might indicate a lack of awareness about mental health challenges and the benefits of seeking professional help. This highlights the complexity of the help-seeking process and the potential influence of other factors on help-seeking intention. Furthermore, it suggests a gap between the theoretical importance of perceived need and the actual recognition of this need among a significant proportion of health workers.

The role of perceived stigma barriers as a negative predictor of help-seeking intention is consistent with other research across various professions and cultural contexts, such as a study on German health workers [22] and American mental health counsellors [33]. Similar stigma-related barriers and reduced professional help-seeking have also been reported in Asian and other LMIC settings, suggesting that sociocultural norms and system-level constraints may amplify concerns about disclosure and confidentiality compared with many Western settings. For example, comparable patterns have been observed in studies from Korea, China, and Ethiopia, where stigma and attitudes consistently shape help-seeking behaviour [36–38]. Indeed, stigma’s negative influence on help-seeking intention extends across various professions, as evident in studies among Canadian public safety personnel and school counsellors [19,21]. These findings highlight the powerful impact of stigma on help-seeking intentions, regardless of the population or profession involved. It highlights the need for interventions to reduce stigma-related barriers, such as stigma reduction campaigns and promoting positive attitudes towards mental health help-seeking.

In our study, variables such as age, sex, income, marital status, and level of education were not significant predictors of help-seeking intention. This contrasts with some previous research that identified these factors as significant predictors [18,38–40]. This discrepancy could be caused by the differences in the sample populations in which the studies were conducted, or the method used to measure help-seeking intention. The specific scales used to measure intention that differs between studies also potentially affected the results. Future research could explore these discrepancies further, perhaps by using consistent measures of intention across different studies and contexts.

### Implications for practice and policy

This study provides a unique understanding of mental health help-seeking intention and its predictors among health workers in Malaysia. These findings have several important implications. From a practice perspective, these findings highlight that interventions are needed to increase health workers’ recognition of their own mental health needs and reduce stigma-related barriers to seeking help. Such interventions to promote mental health help-seeking among health workers might also focus on the common barriers perceived in this study. Healthcare providers should also consider implementing programs and interventions to reduce stigma, increase awareness about the importance of mental health services, and ensure confidentiality. These efforts could include educational workshops, peer support groups, and awareness campaigns designed to promote help-seeking and support among health workers [41,42].

From a policy perspective, our findings emphasise the importance of creating a supportive work environment. This involves not only creating structures that facilitate help-seeking but also strengthening policies that prioritise mental health among health workers and actively working to diminish stigma within healthcare settings. Leaders and decision-makers in health care services should lead by example to minimise the stigma among health workers by promoting a workplace culture of openness, trust, respect, and support around mental health problems. Normalising conversations related to psychological issues might help alleviate some stigma among colleagues [6].

### Study limitations and opportunities for future research

Although this study has contributed new knowledge to the field, it must be interpreted cautiously due to a variety of limitations. First, because this research was conducted using a cross-sectional methodology, it restricts our ability to draw any conclusions about the possible causal inferences between the variables. Second, the unequal distribution of the ethnic group and gender distribution of the respondents in this study could potentially affect the generalizability of these findings. However, this is to be expected because the study was conducted in a population area where most people were Malay. Third, the data collection mainly relied on self-report measures, which are prone to social desirability bias since people tend to report thoughts that they think others want to hear. However, self-reporting was necessary for this study due to its focus on personal perceptions. Fourth, a potential limitation of this study is non-response bias. It is possible that those most inclined to seek help or with a more favorable attitude toward mental health services were more likely to participate, which may limit the generalizability of our findings. For example, the discrepancy between the number of individuals with a formal mental health diagnosis (14) and those who reported seeking help (51) could be due to underreporting or misunderstanding of the diagnosis question. This discrepancy highlights the need for further investigation into how mental health is perceived and diagnosed among healthcare workers

With these limitations, future research could use a longitudinal study design to examine the temporal relationships between help-seeking intention, perceived need for help, and perceived stigma barriers. It would also be beneficial to include a more balanced gender and ethnic distribution to explore potential differences in help-seeking intentions and perceived barriers. This would not only enhance the generalizability of the findings but also allow for exploring potential differences in the results across different demographic groups. Understanding these differences may aid in designing targeted interventions to promote help-seeking among specific groups of health workers.

Additionally, future studies might consider incorporating qualitative techniques such as in-depth interviews or focus group discussions to further understand health workers’ help-seeking intentions. Given the results from this study, we believe that more studies using qualitative methods should be conducted to better understand why some health workers had lower intentions to seek help. Qualitative research methods, such as in-depth interviews, could be useful in exploring the complex social and cultural factors that contribute to this phenomenon.

Despite these limitations, the findings of this study still provide useful insights into help-seeking intentions and their predictors among health workers in the East Coast Region of Peninsular Malaysia. The findings highlight the importance of addressing the perceived need for help and stigma-related barriers in efforts to promote mental health help-seeking among health workers. Therefore, future intervention studies should consider these factors to improve the mental health of health workers.

## Conclusions

In conclusion, this study provides evidence that perceived need for help and stigma-related barriers are key predictors of professional mental health help-seeking intention among health workers in Pahang (East Coast of Peninsular Malaysia). These findings support practical and policy actions within healthcare organizations, including: (i) strengthening confidential and easily accessible pathways to mental health support (e.g., clear referral routes and private access), (ii) implementing workplace stigma-reduction and mental health literacy initiatives, and (iii) improving organizational conditions that enable help-seeking, such as protected time or flexible arrangements to attend appointments. Future research should evaluate targeted interventions addressing these barriers and assess whether changes in workplace systems translate into improved help-seeking behaviours [41].

## References

[1] Dutheil F, Aubert C, Pereira B, Dambrun M, Moustafa F, Mermillod M, et al. Suicide among physicians and health-care workers: A systematic review and meta-analysis. PLoS One. 2019;14(12):e0226361. doi: 10.1371/journal.pone.0226361 31830138 PMC6907772

[2] Eyu Zhen ST, Madeehah T, Mohd TA, Mohamad Ismail SM, Chai WG, Chin TF, et al. Mental health status of healthcare workers in primary health clinics in Sepang. Malaysian Journal of Psychiatry. 2020;29(2).

[3] Spoorthy MS, Pratapa SK, Mahant S. Mental health problems faced by healthcare workers due to the COVID-19 pandemic-A review. Asian J Psychiatr. 2020;51:102119. doi: 10.1016/j.ajp.2020.102119 32339895 PMC7175897

[4] Woon LS-C, Tiong CP. Burnout, Mental Health, and Quality of Life Among Employees of a Malaysian Hospital: A Cross-sectional Study. Ann Work Expo Health. 2020;64(9):1007–19. doi: 10.1093/annweh/wxaa075 32918467

[5] Muller AE, Hafstad EV, Himmels JPW, Smedslund G, Flottorp S, Stensland SØ, et al. The mental health impact of the covid-19 pandemic on healthcare workers, and interventions to help them: A rapid systematic review. Psychiatry Res. 2020;293:113441. doi: 10.1016/j.psychres.2020.113441 32898840 PMC7462563

[6] Søvold LE, Naslund JA, Kousoulis AA, Saxena S, Qoronfleh MW, Grobler C, et al. Prioritizing the Mental Health and Well-Being of Healthcare Workers: An Urgent Global Public Health Priority. Front Public Health. 2021;9:679397. doi: 10.3389/fpubh.2021.679397 34026720 PMC8137852

[7] Vizheh M, Qorbani M, Arzaghi SM, Muhidin S, Javanmard Z, Esmaeili M. The mental health of healthcare workers in the COVID-19 pandemic: A systematic review. J Diabetes Metab Disord. 2020;19(2):1967–78. doi: 10.1007/s40200-020-00643-9 33134211 PMC7586202

[8] Henderson C, Evans-Lacko S, Thornicroft G. Mental illness stigma, help seeking, and public health programs. Am J Public Health. 2013;103(5):777–80. doi: 10.2105/AJPH.2012.301056 23488489 PMC3698814

[9] Fridner A, Belkić K, Marini M, Gustafsson Sendén M, Schenck-Gustafsson K. Why don’t academic physicians seek needed professional help for psychological distress?. Swiss Med Wkly. 2012;142:w13626. doi: 10.4414/smw.2012.13626 22802214

[10] Grover S, Dua D, Shouan A, Nehra R, Avasthi A. Perceived stress and barriers to seeking help from mental health professionals among trainee doctors at a tertiary care centre in North India. Asian J Psychiatr. 2019;39:143–9. doi: 10.1016/j.ajp.2018.12.020 30639973

[11] Jones N, Whybrow D, Coetzee R. UK military doctors; stigma, mental health and help-seeking: a comparative cohort study. J R Army Med Corps. 2018;164(4):259–66. doi: 10.1136/jramc-2018-000928 29523754

[12] She R, Wang X, Zhang Z, Li J, Xu J, You H, et al. Mental Health Help-Seeking and Associated Factors Among Public Health Workers During the COVID-19 Outbreak in China. Front Public Health. 2021;9:622677. doi: 10.3389/fpubh.2021.622677 34046387 PMC8144452

[13] Clough BA, March S, Leane S, Ireland MJ. What prevents doctors from seeking help for stress and burnout? A mixed-methods investigation among metropolitan and regional-based australian doctors. J Clin Psychol. 2019;75(3):418–32. doi: 10.1002/jclp.22707 30431644

[14] Edwards JL, Crisp DA. Seeking help for psychological distress: Barriers for mental health professionals. Australian Journal of Psychology. 2017;69(3):218–25. doi: 10.1111/ajpy.12146

[15] Wijeratne C, Johnco C, Draper B, Earl J. Doctors’ reporting of mental health stigma and barriers to help-seeking. Occup Med (Lond). 2021;71(8):366–74. doi: 10.1093/occmed/kqab119 34534344

[16] White MM, Clough BA, Casey LM. What do help-seeking measures assess? Building a conceptualization framework for help-seeking intentions through a systematic review of measure content. Clin Psychol Rev. 2018;59:61–77. doi: 10.1016/j.cpr.2017.11.001 29153743

[17] Yu Y, Liu Z, Hu M, Liu H, Yang JP, Zhou L, et al. Mental Health Help-Seeking Intentions and Preferences of Rural Chinese Adults. PLoS One. 2015;10(11):e0141889. doi: 10.1371/journal.pone.0141889 26545095 PMC4636424

[18] Cohen D, Winstanley SJ, Greene G. Understanding doctors’ attitudes towards self-disclosure of mental ill health. Occup Med (Lond). 2016;66(5):383–9. doi: 10.1093/occmed/kqw024 27030052 PMC4913366

[19] Krakauer RL, Stelnicki AM, Carleton RN. Examining Mental Health Knowledge, Stigma, and Service Use Intentions Among Public Safety Personnel. Front Psychol. 2020;11:949. doi: 10.3389/fpsyg.2020.00949 32547443 PMC7273931

[20] Keene CN. Predicting help-seeking attitudes and intentions in a Diné sample. In: Denver UO, editor. 2018.

[21] Mullen PR, Crowe A. Self‐Stigma of Mental Illness and Help Seeking Among School Counselors. Jour of Counseling & Develop. 2017;95(4):401–11. doi: 10.1002/jcad.12155

[22] Wulf IC. Help-seeking and help-outreach intentions of healthcare workers—the role of mental health literacy and stigma in the workplace. Front Educ. 2022;7. doi: 10.3389/feduc.2022.856458

[23] Hanafiah AN, Van Bortel T. A qualitative exploration of the perspectives of mental health professionals on stigma and discrimination of mental illness in Malaysia. Int J Ment Health Syst. 2015;9:10. doi: 10.1186/s13033-015-0002-1 25774215 PMC4359579

[24] Pendit UC, Koo AC. Openness towards mental illness in Malaysia. Malaysia UK. 2020.

[25] Sum VLW, Sabran MS, Adam AMIS, Seng LC. Factors influence intention to seek counselling service among health professionals: application of theory of planned behavior. International Journal of Humanities & Social Science Studies (IJHSSS). 2015;2(2):87–95.

[26] Khairudin R. Mental health help-seeking and access to services among schooling adolescent in Selangor: A mixed method study. In: Malaya U, editor. Universiti of Malaya; 2019.

[27] Hammer JH, Spiker DA. Dimensionality, reliability, and predictive evidence of validity for three help-seeking intention instruments: ISCI, GHSQ, and MHSIS. J Couns Psychol. 2018;65(3):394–401. doi: 10.1037/cou0000256 29672088

[28] Kunyahamu MS, Daud A, Tengku Ismail TA, Md Tahir MF. Translation, Adaptation, and Validation of the Malay Version of the Barriers to Access to Care Questionnaire for Assessing the Barriers to Seeking Mental Health Care Among the Health Workforce in the East Coast Region of Peninsular Malaysia. Cureus. 2023;15(7):e41405. doi: 10.7759/cureus.41405 37546078 PMC10402845

[29] IBM SPSS Statistics for Windows. IBM Corp; 2019.

[30] Aruta JJBR, Maria A, Mascarenhas J. Self-compassion promotes mental help-seeking in older, not in younger, counselors. Curr Psychol. 2022;:1–11. doi: 10.1007/s12144-022-03054-6 35400980 PMC8976458

[31] Nation U. The pandemic accelerant: How COVID-19 advanced our mental health priorities. 2021. [Cited 2024 September 25]. https://www.un.org/en/un-chronicle/pandemic-accelerant-how-covid-19-advanced-our-mental-health-priorities

[32] Chong HX, Hashim AH, Osman S. Civil servants’ intention in seeking counselling services. Jurnal Pengguna Malaysia. 2018;31.

[33] Miller CA. Help Seeking for Mental Health Problems in Mental Health Counselors: Prevalence, Barriers, and Predictors. In: Louis U-S, editor. University of Missouri-St. Louis. 2020.

[34] Gold KJ, Andrew LB, Goldman EB, Schwenk TL. “I would never want to have a mental health diagnosis on my record”: A survey of female physicians on mental health diagnosis, treatment, and reporting. Gen Hosp Psychiatry. 2016;43:51–7. doi: 10.1016/j.genhosppsych.2016.09.004 27796258

[35] Tay S, Alcock K, Scior K. Mental health problems among clinical psychologists: Stigma and its impact on disclosure and help-seeking. J Clin Psychol. 2018;74(9):1545–55. doi: 10.1002/jclp.22614 29573359

[36] Saint Arnault DM, Gang M, Woo S. Factors Influencing on Mental Health Help-seeking Behavior Among Korean Women: A Path Analysis. Arch Psychiatr Nurs. 2018;32(1):120–6. doi: 10.1016/j.apnu.2017.10.003 29413062

[37] Yeshanew B, Belete A, Necho M. Help-seeking intention and associated factors towards mental illness among residents of Mertule Mariam town, East Gojam Zone, Amhara Region, Ethiopia: a mixed-method study. Ann Gen Psychiatry. 2020;19:12. doi: 10.1186/s12991-020-00261-y 32127907 PMC7045738

[38] Chin WY, Chan KTY, Lam CLK, Lam TP, Wan EYF. Help-seeking intentions and subsequent 12-month mental health service use in Chinese primary care patients with depressive symptoms. BMJ Open. 2015;5(1):e006730. doi: 10.1136/bmjopen-2014-006730 25631313 PMC4316433

[39] Muhamad Ramzi NSA, Deady M, Petrie K, Crawford J, Harvey SB. Help-seeking for depression among Australian doctors. Intern Med J. 2021;51(12):2069–77. doi: 10.1111/imj.15035 32833296

[40] Seyfi F, Poudel KC, Yasuoka J, Otsuka K, Jimba M. Intention to seek professional psychological help among college students in Turkey: influence of help-seeking attitudes. BMC Res Notes. 2013;6:519. doi: 10.1186/1756-0500-6-519 24313965 PMC4028976

[41] Ramírez-Vielma R, Vaccari P, Cova F, Saldivia S, Vielma-Aguilera A, Grandón P. Interventions to reduce the stigma of mental health at work: a narrative review. Psicol Reflex Crit. 2023;36(1):14. doi: 10.1186/s41155-023-00255-1 37213032 PMC10203079

[42] Shim YR, Eaker R, Park J. Mental Health Education, Awareness and Stigma Regarding Mental Illness Among College Students. J Ment Health Clin Psychol. 2022;6(2):6–15. doi: 10.29245/2578-2959/2022/2.1258

